# Host Cell Wall Damage during Pathogen Infection: Mechanisms of Perception and Role in Plant-Pathogen Interactions

**DOI:** 10.3390/plants10020399

**Published:** 2021-02-19

**Authors:** Riccardo Lorrai, Simone Ferrari

**Affiliations:** Dipartimento di Biologia e Biotecnologie “Charles Darwin”, Sapienza Università di Roma, Piazzale Aldo Moro 5, 00185 Rome, Italy; riccardo.lorrai@uniroma1.it

**Keywords:** plant cell wall, plant-pathogen interactions, Damage-Associated Molecular Patterns, cell wall-degrading enzymes, plant innate immunity

## Abstract

The plant cell wall (CW) is a complex structure that acts as a mechanical barrier, restricting the access to most microbes. Phytopathogenic microorganisms can deploy an arsenal of CW-degrading enzymes (CWDEs) that are required for virulence. In turn, plants have evolved proteins able to inhibit the activity of specific microbial CWDEs, reducing CW damage and favoring the accumulation of CW-derived fragments that act as damage-associated molecular patterns (DAMPs) and trigger an immune response in the host. CW-derived DAMPs might be a component of the complex system of surveillance of CW integrity (CWI), that plants have evolved to detect changes in CW properties. Microbial CWDEs can activate the plant CWI maintenance system and induce compensatory responses to reinforce CWs during infection. Recent evidence indicates that the CWI surveillance system interacts in a complex way with the innate immune system to fine-tune downstream responses and strike a balance between defense and growth.

## 1. Introduction

**Figure 1:**
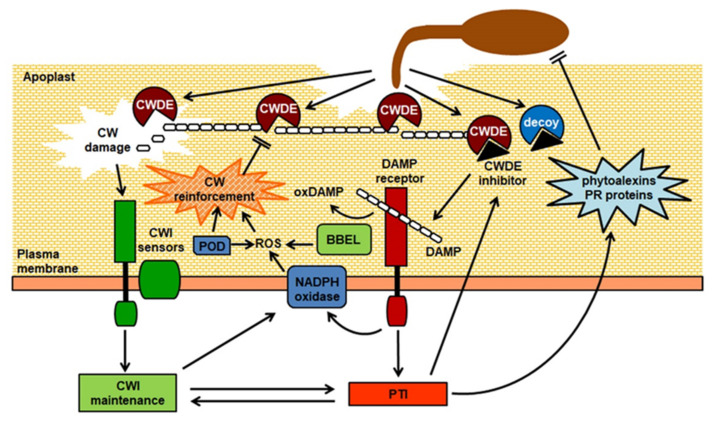
Overview of responses induced by cell wall damage during pathogen infection. CW, cell wall; CWDE, cell wall-degrading enzyme; CWI, cell wall integrity; PTI, pattern-triggered immunity; DAMP, Damage-Associated Molecular Pattern; BBEL, berberine bridge enzyme-like protein; oxDAMP, oxidized DAMP; POD, peroxidase; ROS, reactive oxygen species. CWDEs secreted by the pathogen degrade CW structural polysaccharides. CW damage activates the CWI perception system, mediated by dedicated sensors, triggering CWI maintenance responses, that include production of ROS mediated by membrane NADPH oxidases and apoplastic PODs, and reinforcement of the CW. Host-encoded inhibitors reduce CWDE activity, slowing down CW degradation and promoting the accumulation of CW-derived DAMPs. Perception of DAMPs by membrane-localized receptors in turn activates PTI, which leads to antimicrobial defence responses, including production of phytoalexins, PR proteins and ROS, that contribute to restrict infection. Negative and positive crosstalk between CWI and PTI fine-tune defence responses triggered by CW damage. DAMPs can be inactivated by apoplastic BBEL proteins, that oxidize CW-derived oligosaccharides, at the same time producing ROS. Some pathogens can secrete decoy proteins that bind to CWDE inhibitors.

All plant cells are surrounded by a stiff but extensible extracellular matrix, the cell wall (CW), that performs different crucial mechanical, biochemical, and physiological functions [1,2,3]. It is now understood that the CW is a complex and plastic structure, whose composition and architecture widely varies among species and within tissues and cells of the same organism, and is extensively re-modelled during growth and development and in response to environmental cues [2]. The major load-bearing component of plant CWs is cellulose, which provides tensile strength; non-cellulosic polysaccharides, structural proteins, and other non-saccharide components, like lignin, all contribute to the specific mechanical and biochemical properties of the CW in different cell types [2,3]. Thanks to their tensile strength, plant CWs provide mechanical support to the cell, permitting elevated internal turgor pressures and modulating cell expansion, ultimately determining cell shape and size [4]. They mediate several additional important functions, including cell adhesion and cell-to-cell communication and, being in contact with the external environment, also provide a chemical-physical barrier to the loss of water and to the attack of pathogenic microorganisms and herbivores [5]. For these reasons, it is not surprising that plant CWs evolved to be extremely resistant to mechanical damage and to enzymatic deconstruction. Many pathogens, to gain access to the host cells, need to either bypass these structures, penetrating through natural openings (e.g., stomata), wounds, or with the aid of vectors, or they employ an arsenal of CW-degrading enzymes (CWDEs) to deconstruct the structural components of the host CW, assisting penetration and diffusion in the host tissues, at the same time providing carbon sources and promoting leakage of nutrients from the protoplast [6]. Host CW degradation can be massive, as in the case of the infection with necrotrophic pathogens, in particular soft-rot agents, that secrete large amounts of CWDEs in the infected tissues, or it can be more localized and controlled, as in the case of biotrophic pathogens that need to keep their host alive and often utilize specialized feeding structures that required extensive remodeling of the host CWs [7]. Increasing evidence indicates the existence of multiple mechanisms that plant cells employ to detect in a timely manner changes in CW integrity (CWI), and to mount responses that compensate for the damage inflicted by the pathogen, stiffening the CW and making it more recalcitrant to deconstruction [8,9,10,11]. This CWI surveillance system also activates signaling pathways that modulate both growth- and defense-related signaling pathways to ensure an effective antimicrobial response with a minimum fitness cost [8,10] (Figure 1). We will provide a summary here of our current knowledge on how microbes degrade plant CW structural components and of the mechanisms employed by plants to counteract this deconstruction and to perceive changes in CWI to regulate immunity.

## 2. Degradation of Host Cell Wall Structural Components during Plant-Pathogen Interactions

The importance of host CW degradation during plant–pathogen interactions is high-lighted by the significant expansion of CWDEs during microbial evolution. Fungi, compared to other taxonomic groups, secrete a remarkable variety of CWDEs [12], though CW degradation can play a major role in virulence also for some bacteria [13,14]. This enzymatic arsenal encompasses carbohydrate-active enzymes (CAZymes) [15] that contribute to various extents to the deconstruction of the host CW in concert with other enzymes required to remove modifications and cleave side chains and intra- and intermolecular bonds [16]. CAZymes with CW-degrading activity are classified as glycoside hydrolases, polysaccharide lyases), and carbohydrate esterases [17]. Notably, mycorrhizal fungal genomes encode fewer CWDEs and CW-modifying proteins than phytopathogen genomes [18], supporting the hypothesis that they have specifically expanded in phytopathogens as a consequence of the selective pressure posed by the plant CW complexity and recalcitrance to degradation.

The diversity of CWDEs reflects the structural complexity of the plant CW as well as the lifestyle of the pathogen [19]. In some cases, direct evidence of the importance of specific enzymes in virulence was obtained from mutants deleted for the corresponding gene(s) or through RNA interference (RNAi) [20,21,22,23]. However, in many cases, gene inactivation was unsuccessful to demonstrate the contribution of specific CWDEs as key virulence factors, probably due to functional redundancy with other enzymes of the same protein family or of other families, that can mask the effects of single or even multiple knock-out mutations [24,25]. The next paragraphs will describe the major plant CW structural components, the microbial enzymes involved in their degradation, and the available evidence supporting their importance in pathogenesis.

### 2.1. Cellulose

Cellulose, the major load-bearing component of plant CWs, is a β-1,4-D-glucan polymer synthesized at the plasma membrane by cellulose synthases (CESAs), processive family-2 glycosyltransferases that simultaneously catalyze D-Glucose (Glc) transfer from UDP-D-Glc to the C4-hydroxyl end of the cellulose polymer and translocate the polysaccharide through the plasma membrane [26,27]. It has been proposed that one trimeric CESA complex produces three glucan chains, forming a protofibril that interacts with five more protofibrils to originate a microfibril composed of 18 cellulose chains [27]. Van der Waals and hydrogen bonds facilitate parallel stacking of multiple β-1,4-D-glucan chains, that can be arranged in ordered or disordered regions, respectively named crystalline or amorphous cellulose [28], the latter resulting in a less rigid structure that is more accessible to water and cellulolytic enzymes, or cellulases [29]. Cellulases can be classified into three major types according to their mode of hydrolysis and substrate specificity [30]. β-1,4-Endoglucanases (EGs; EC 3.2.1.4) hydrolyze the internal bonds of the cellulose chains in the amorphous regions, producing new chain ends, whereas cellobiohydrolases (CBHs; EC 3.2.1.91) are exoglucanases that attach to cellulose reducing- or non-reducing ends, both in crystalline and amorphous parts, and hydrolyze it into cellobiose units [30], which are finally cleaved into Glc monomers by β-glucosidases (EC 3.2.1.21) [31]. This clear-cut distinction is now challenged by the notion that cellulases have evolved overlapping modes of action, ranging from totally random EGs through processive EGs to strictly exo-acting, highly processive CBHs [16]. In contrast to fungi and bacteria, most metazoans lack endogenous cellulases, and rely on symbiotic microorganisms to digest cellulose, apart from some insects and nematodes [32,33,34].-EGs identified in the gut of the nematodes *Globodera rostochiensis* and *Heterodera glycines* were the first CWDEs encoded by animal genomes to be discovered [32]. Cellulases are expressed during infection in several pathosystems, and they are thought to contribute to virulence, though direct evidence for their role in pathogenicity is limited, compared to other CWDEs. Disruption of cellulase genes in phytopathogenic microorganisms often failed to impair virulence [21,35]. This may reflect an elevated functional redundancy of the different cellulases secreted by pathogens during infection, making it difficult to observe a phenotype in single knock-out mutants. However, it has been demonstrated that cellulases contribute to host penetration and tissue invasion in *Magnaporthe grisea* [36] and to root penetration in *G. rostochiensis* [34], and are major virulence factors in some bacteria, like *Clavibacter michiganensis,* the causal agent of tomato wilt [37]. In addition to glucanases, lytic polysaccharide monooxygenases help digest inaccessible crystalline cellulose [38]. These enzyme are found in different Ascomycota and Basidiomycota, but their importance in plant–pathogen interactions is not yet established [39].

### 2.2. Pectins

Pectins are very complex polymers that are characterized by the presence of acidic sugar moieties and that vary in composition and organization among different plant species and in different tissues [40]. Main pectins are homogalacturonan (HG), rhamnogalacturonan I (RGI), rhamnogalacturonan II (RGII), and xylogalacturonan (XGA). HG is a homopolymer of α-(1→4)-linked D-galacturonic acid (GalA), which is synthesized in the Golgi apparatus in a completely methylesterified form and undergoes in muro demethylesterification by pectin methylesterases (PMEs, E.C. 3.1.1.11) [41]. RGII is far more complex than HG, being constituted by an HG backbone substituted with complex side chains containing 12 different sugars, whereas RGI has a disaccharide backbone of GalA and L-Rhamnose (Rha), and its structure is highly variable according to cell types and developmental stages [40]. Many microorganisms secrete a wide range of enzymes able to modify and degrade pectins to monomers, mostly GalA and Rha, that can be uptaken and utilized as carbon sources [42]. Pectinases are classified according to their site of cleavage, (endo- and exo-pectinases, if they cleave within or at the end of the substrate chain, respectively), preferred substrate (pectin or polygalacturonic acid, commonly named pectate) and the mode of cleavage of the glycosidic bond (hydrolases or lyases) [43]. Polygalacturonases (PGs) catalyze the hydrolytic cleavage of glycosidic α(1-4) linkages on HG, either internally (endo-PGs, E.C.3.2.1.15), releasing oligomers and monomers of GalA, or at the non-reducing end of the HG chain (exo-PGs, E.C.4.2.1.67 and E.C.4.2.1.82). PGs are usually only active on non-esterified pectin regions, thus requiring the removal of methylester groups by PMEs for complete depolymerization [43]. Pectin lyases (PNLs, E.C.4.2.2.10) act via β-elimination on highly methyl-esterified pectins and do not require calcium for enzymatic activity, whereas pectate lyases (endo-PLs, E.C.4.2.2.2 and exo-PLs, E.C.4.2.2.9) cleave non-esterified GalA residues and require calcium for optimal activity [44]. PLs represent the largest group of bacterial pectinolytic enzymes, whereas PGs are the most prevalent pectinases secreted by fungi [13,45].

The importance of pectin degradation during plant–pathogen interactions was suggested more than 30 years ago [46], and pectinolytic enzymes are currently thought to contribute to virulence in several pathosystems [45]. Pectinase activity can deconstruct the plant middle lamella that is responsible for cell-to-cell adhesion, leading to maceration of the host tissues, which is most significant in soft rot-causing microbes but appears to assist invasion in a wide range of pathogens [45]. There is indeed evidence that differential sets of pectinases are recruited under varying physiological conditions—e.g., saprophytic versus pathogenic growth—and play different roles in pathogenesis [45]. Pectinases are among the first enzymes to be secreted by many phytopathogens, indicating that their activity is essential for subsequent degradation of other CW structural components [47]. This hypothesis is supported by the observation that expression of a fungal PG in Arabidopsis and tobacco facilitates cellulose degradation by cellulases [48]. Gene deletion experiments confirmed the crucial role of pectinases as virulence factors for several pathogens. For instance, deletion of the PG *pecA* gene in *Aspergillus flavus* reduces lesion development in cotton, and expression of the same gene in a strain that lacks PG increases the size of lesions [49]. A single PG is required for full virulence of *Alternaria citri* on citrus fruit [22], and a *Claviceps purpurea* strain carrying a deletion of two PG genes is nearly nonpathogenic on rye [50]. The importance of pectinases is more evident in the case of necrotrophic pathogens that cause soft rot symptoms. For instance, *B. cinerea* produces a variety of pectinases, including exo- and endo-PGs, PMEs, PNLs, and PLs [42], of which PGs are probably those best characterized. The *B. cinerea* genome codes for six endoPGs (BcPG1-6), the expression of which varies depending on the infected tissues [20,51]. BcPG1 and BcPG2 can induce tissue collapse and necrosis and play a role in virulence on tomato, apple, broad bean, and Arabidopsis [20,52]. The availability of a set of endoPGs with slightly different characteristics in terms of substrate specificity might enable a pathogen to hydrolyze a larger spectrum of pectin types from different host species. However, a *B. cinerea* knockout mutant for BcPG1 shows loss of virulence on different hosts [20], indicating that specific PG isoforms can play a preeminent role in pathogenesis.

In addition to PGs, other pectinolytic activities can contribute to pathogenesis. The importance of PLs in pathogenicity is mostly acknowledged for Pectobacteria [14]. Down-regulation of PLs in *H. schachtii* indicates that these enzymes are also necessary for nematode root invasion [53]. The importance of other pectinolytic activities in plant-pathogen interactions is not so clear. An *A. tubingensis* XGA hydrolase (XGH) specifically acts on XGA by cleaving the GalA backbone in an endo-manner [54]. This enzyme belongs to the pectin degrading glycoside hydrolase family 28, which includes PGs, and XGA can also be degraded by exo-PGs [55]. Reports on the microbial degradation system for RG-I and RG-II are also limited, possibly due to the complex structure of this polysaccharide, although a few RG hydrolases and lyases from fungi and bacteria have been identified [56,57,58,59]. It must be noted that the activity of microbial pectinases is strictly dependent on the status of methylation of their substrates, and many pathogens secrete PMEs to remove methyl groups from pectin, making it more prone to attack by PGs and PLs and contributing to virulence [60]. Pathogens can also hijack host PMEs to assist infection, as suggested by the observation that *P. carotovorum* and *B. cinerea* induce in Arabidopsis a rapid expression of the host-encoded AtPME3, that acts as a susceptibility factor and is required for the initial colonization of the host tissues [61].

### 2.3. Hemicelluloses

Hemicelluloses are a heterogeneous group of neutral polysaccharides that includes xylans, xyloglucans, mannans, glucomannans, and β-(1→3,1→4)-glucans [62]. Xylans have a β-1,4-linked D-xylose (Xyl) backbone, which is commonly substituted with 4-*O*-methyl-glucuronosyl residues at O-2 position or with arabinose (Ara) residues at O-2 or O-3 position. Xyloglucans, like cellulose and xylans, have a β-1,4-glucan backbone that can be unbranched or substituted with α-(1→6)-D-Xyl. Xyl residues can be further substituted at O-2 with Ara or galactose (Gal), and Gal residues can be further substituted with fucose (Fuc). The degree of substitution of the glucan backbone varies among taxonomic groups and confers specific properties, such as solubility and anionic behavior, to different xyloglucans [63]. In mannans and glucomannans, the backbone is a β-(1→4)-linked polysaccharide consisting, respectively, of D-mannose (Man) or of Man and Glc. Mannans and glucomannans have side chains of single Gal residues bound to Man with a α-(1→6) glycosidic bond. Little direct evidence is available for hemicellulases as virulence determinants in plant pathogens. One example is the xylanase Xyn11A of *B. cinerea*, whose disruption causes a 30% decrease in extracellular xylanase activity, but reduces average lesion size by more than 70% [64], possibly because the mutant loses its ability to induce necrosis in the host tissues. In *M. oryzae*, silencing of different xylanases resulted in greater defects in virulence as compared to knockdowns of cellulases [23]. A functional xylanase has been identified in *Meloidogyne incognita* [65], suggesting that xylanases might be virulence factors also for nematodes.

### 2.4. Lignin and Other Cell Wall Structural Components

An important component of plant immunity is the activation of responses aimed at the reinforcement of the host CW, like deposition of lignin. Lignin is a hydrophobic polymer of monolignols synthesized in the cytoplasm and transported to the apoplast, where they are oxidatively polymerized by plant class III peroxidases (PODs) and laccases [66]. Secondary CWs can be extensively lignified in specialized tissues, like xylem vessels, but lignification also occurs in non-specialized cell types in response to mechanical damage or pathogen infection, stiffening the CW and restricting enzymatic degradation of other structural components [67]. Secretion of PODs in response to microbial attack can contribute to CW reinforcement by mediating lignin polymerization [68]. Deposition of lignin was associated with resistance of cotton to *Verticillium dahliae* [69] and of *Camelina sativa* to *Sclerotinia sclerotiorum* [70]. Different microbes secrete ligninolytic enzymes, including phenol oxidases (laccases) and heme-containing lignin, manganese, and multifunctional PODs; however, only Basidiomycota causing white rots can completely degrade lignin [71], though bacteria able to break down lignin have been reported [72]. Wood-degrading fungi mostly live as saprotrophs or weak parasites, but laccase production occurs in some phytopathogenic ascomycetes such as *Gaeumannomyces graminis* [73] and *M. grisea* [74]. However, direct evidence of the role of these enzymes in host penetration or invasion is not available. Notably, plant PODs also mediate the formation of cross-links between phenolic compounds and between polysaccharides and phenolics, such as ferulic acid [75], enhancing CWs recalcitrance to enzymatic degradation and resistance to fungi [76]. Fungal ferulic acid esterases may indeed contribute to virulence in some pathosystems [77]. Other hydrophobic compounds can be found in the CW of specific cell types, most notably cutin and waxes in the cuticle of the epidermal cells of aerial organs [78]. The microbial enzymes involved in their degradation and their role in plant-pathogen interactions have been recently reviewed by other authors [79] and will not be discussed here.

Plant CWs contain structural proteins with a broad range of functions. Extensins, the first class of structural CW proteins identified in plants [80], are hydroxyproline- (Hyp-) rich glycoproteins (HRGPs), where the addition of arabino-oligosaccharides to the core polypeptide induces an extended polyproline-II helical structure giving the protein a rod-like shape. Extensins in dicots are rich in Ser as well as Hyp, and usually show a pentapeptide repeat motif, Ser-(Hyp)_3-4_, interspersed with amino acid residues (e.g., Tyr and Lys) that are important for POD-mediated cross-linking. Tyr residues can cross link with each other, creating intra- and intermolecular bridges to form a protein network believed to stabilize or reinforce the CW in cells that have stopped expansion, or in response to stress [81,82]. During pathogenesis, polymerization of extensins can stiffen CWs, delaying pathogen colonization [83,84]. Some reports suggest that metalloproteases are directly involved in the degradation of CW structural proteins, such as extensins [85,86], but their role in pathogenesis is still far from being completely understood.

### 2.5. Inhibitors of CWDEs

Beside reinforcing the CW, as a countermeasure to protect it from degradation, plants can deploy extracellular proteins that bind to and inhibit microbial CWDEs [87,88]. The most extensively studied of such inhibitors are PG-inhibiting proteins (PGIPs), first identified in 1971 by Albersheim and colleagues [89]. PGIPs are found in all plant species so far analyzed and inhibit PGs secreted by bacteria, fungi, nematodes, and insects [90,91]. They belong to the leucine-rich repeat (LRR) protein family, with a negatively charged surface on the concave side of the protein that is crucial for the interaction and inhibition of PGs [92,93]. In addition to hampering pectin degradation, the interaction between PGs and PGIPs during infection is thought to promote the formation of oligogalacturonides (OGs), that can induce a variety of defense responses in the host [94], as further discussed below. Genome analysis has shown that PGIPs are encoded by single genes or small gene families, whose members can show functional redundancy and sub-functionalization [91]. Different PGIP isoforms from a plant species can exhibit different activities against PGs from different fungi or even different PGs from the same fungal strain. For instance, in *Phaseolus vulgaris*, PvPGIP2 inhibits PGs from both *Fusarium moniliforme* and *A. niger*, whereas PvPGIP1 is effective only against the *A. niger* enzyme [93]. The Arabidopsis genome carries two tandemly repeated *PGIP* genes, *AtPGIP1* and *AtPGIP2*, encoding closely related inhibitors with similar activity against BcPG1 of *B. cinerea* but different activity against a PG from *Colletotrichum acutatum* [95]. Expression of these two genes during fungal infection is mediated by different signaling pathways, since *AtPGIP2* expression is regulated by jasmonates (JAs), whereas *AtPGIP1* expression is induced by OGs independently of these phytohormones [95]. This suggests that duplication of *PGIP* genes followed by sub-functionalization might have an adaptive significance for combating different pathogens more efficiently. Direct evidence indicates that PGIPs have a protective effect against pathogens; for instance, overexpression of *PGIP* genes increases resistance to *B. cinerea* in tomato, Arabidopsis, tobacco, and grapevine [95,96,97,98], and to *F. graminearum* and *Bipolaris sorokiniana* in wheat [99,100]. Consistently, suppression of the expression of endogenous PGIPs increases susceptibility to *B. cinerea* in Arabidopsis [101] and to *F. graminearum* in wheat [102].

Many proteins that inhibit fungal xylanases have also been identified, mostly from wheat (*Triticum aestivum*) and rice [87]. Wheat produces two structurally different types of inhibitors, *T. aestivum* xylanase inhibitors (TAXIs) [103] and xylanase-inhibiting proteins (XIPs) [104]. A third type of inhibitors, named thaumatin-like xylanase inhibitors (TLXIs), for their similarity to plant thaumatins, was identified in wheat [105]. TAXIs and XIPs have no sequence homology; the wheat TAXI-I is structurally related to aspartic proteases, though it lacks proteolytic activity [106]. Crystallographic studies revealed a direct interaction between the inhibitor and the xylanase active site in a substrate-mimicking fashion [106]. Xyloglucan-specific endoglucanase inhibitor proteins (XEGIPs), found in some dicots [107,108], appear to inhibit their target via an inhibition loop that mimics the interaction between XEG and its substrate [109]. There is evidence that xylanase inhibitors have a defensive role in pant–pathogen interactions, as shown by the increased resistance to *B. cinerea* in wheat and Arabidopsis plants overexpressing the wheat TAXI-I [110]. In soybean, an inhibitor of the *Phytophthora sojae* xyloglucan endoglucanase PsXEG1, named GmGIP1, binds to and blocks its target. Notably, *P. sojae* in turn has evolved a paralogous decoy protein (PsXLP1) that has no enzymatic activity but interacts more tightly with GmGIP1 than PsXEG1, thus preventing the inhibition of its hydrolytic activity [111]. The production of decoy pseudoenzymes to evade inhibition of CWDEs might be more widespread than currently acknowledged [112].

## 3. Is There Anybody Out There? Perception of Cell Wall Degradation and Activation of Defense Responses

As described in the previous section, enzymatic degradation of the host CWs plays a crucial role in virulence of several phytopathogens. It is therefore not surprising that plants have evolved sophisticated mechanisms to monitor CWI and, in case of damage, ensure the activation of proper compensatory responses aimed at restoring functionality of the CW and at reinforcing it to limit pathogen invasion [113]. Maintenance of CWI must also occur in response to other stresses that affect CW structure and/or functionality, and is of extreme importance during growth and development, as the plant cell needs to continuously monitor the physicochemical status of its wall to balance extensibility with deposition of new material. It is largely acknowledged that, when CWI is altered, multiple stimuli contribute to trigger downstream compensatory responses [9,113]. These stimuli might be of mechanical nature, including membrane stretching and changes in the CW surface tension, leading to conformational changes in dedicated CWI sensors and activation of downstream signaling cascades. Moreover, signaling molecules, most notably CW-derived fragments, released in the apoplast because of CW damage, can act as signaling molecules to trigger compensatory responses. Notably, loss of CWI can also affect the expression of defense responses typically associated to pathogen infection, and recent evidence suggests the existence of cross-talk mechanisms balancing PTI and growth in the presence of CW damage [8,9]. Many recent excellent reviews discuss the molecular mechanisms responsible for CWI maintenance and its role in development and response to abiotic and biotic stresses [8,9,11,113,114]. Here we will summarize this knowledge, with particular focus on its relevance for plant-pathogen interactions.

### 3.1. Cell Wall-Derived Fragments as Elicitors of Defense Responses

The notion that plants could detect CW-derived fragments released by pathogen CWDEs was already proposed at the beginning of the 1980s, based on the observation that an endogenous elicitor of phytoalexins could be extracted from soybean CWs [115]. Subsequent studies demonstrated that OGs, oligomers of α-1,4-linked galacturonosyl residues obtained by partial hydrolysis of polygalacturonic acid, were active as elicitors of defense responses [116]. This led to the hypothesis that degradation of HG during microbial infection causes the accumulation of elicitor-active OGs in the apoplast, triggering downstream defense responses [117]. Research conducted in the following years has in part elucidated the mode of generation of OGs during pathogen attack and the molecular mechanisms underlying their perception and transduction, and have confirmed the role of OGs in defense against pathogens but also in growth and development [94,118,119]. From these studies stemmed the proposal to consider plant CW-derived elicitors as Damage-Associated Molecular Patterns (DAMPs), endogenous molecules that, as also observed in animals, are released from cellular components during pathogen attack or other stresses (e.g., wounding) and are recognized as “non-self” signals that trigger an immune response [118,120].

OGs are probably the best characterized plant DAMPs, and several reports indicate that they elicit in many species a wide range of defense responses, largely overlapping with those induced by microbial Pathogen-Associated Molecular Patterns (PAMPs), including accumulation of phytoalexins, glucanase, and chitinase, deposition of callose, production of reactive oxygen species (ROS), and nitric oxide [94]. OGs are thought to be produced during infection upon partial degradation of HG by microbial PGs [117], but might also be generated by endogenous PGs in response to mechanical damage [121]. OG activity is affected by their degree of polymerization (DP), as OGs with a DP between 10 and 15 are the most effective [122,123], though OGs with a lower DP can also elicit both defense and developmental responses [124,125]. Inhibition of microbial PGs by host PGIPs is thought to favor the accumulation of elicitor-active OGs in the apoplast [126], as confirmed by the observation that OGs are generated in vivo in transgenic Arabidopsis plants expressing a fusion protein between a fungal PGs and a PGIP [127]. On the other hand, it was recently reported that the majority of OGs generated during infection of Arabidopsis leaves with *B. cinerea* are the product of PNLs rather than PGs [124]. These data suggest that multiple pectinases might contribute to the generation of OGs depending on the pathosystem. Activity of OGs generated *in planta* during infection might also be affected by their methylation status. Like microbial PMEs, plant PMEs play an important role in preparing the substrate for processing by microbial and endogenous PGs and PLs [43], and pectin esterification affects plant susceptibility to infections. For example, the degree of methylesterification of pectin in bean cultivars resistant to *C. lindemuthianum* is higher than in near-isogenic susceptible lines [128]. Arabidopsis plants overexpressing a PME inhibitor (PMEI) exhibit a higher pectin degree of methylesterification and are more resistant to *B. cinerea* [129]. It is possible that a highly esterified pectin might serve as a poorer carbon source for the growth of this fungus [129]. However, the esterification status of pectin likely also affects the structure and amount of active OGs released during infection, influencing the outcome of some plant–pathogen interactions. For instance, OGs purified from fruits of transgenic *Fragaria vesca* plants overexpressing a strawberry PME have a reduced degree of esterification, which is necessary to elicit defense responses, and the transgenic plants have constitutively activated pathogen defense responses, resulting in increased resistance to *B. cinerea* [130]. However, the exact role of methylation in the biological activity of OGs is still unclear and deserves further investigation.

Due to the complexity of plant CWs and the multiplicity of microbial CWDEs released during infection, it is expected that, beside OGs, many more CW-derived fragments might act as DAMPs and mediate the activation of defense responses. Degradation of cellulose by pathogen cellulolytic enzymes generates cellooligomers (cellodextrins, CDs), such as cellobiose and cellotriose, that induce a wide range of defense responses [131,132]. Notably, in Arabidopsis, cellobiose triggers a signaling cascade similar to that triggered by OGs but is unable to induce the production of ROS or callose deposition [132], whereas cellotriose and, to a lesser extent, CD 4-6, induce production of ROS, phosphorylation of mitogen-activated protein kinases (MAPKs) and expression of defense genes [133]. Xyloglucan oligosaccharides derived from hemicellulose trigger in grapevine and Arabidopsis a signaling cascade similar to OGs, inducing resistance against pathogens [134]. Other CW-derived oligosaccharides with elicitor activity include a mannan oligosaccharide from galactomannan [135] and an arabinoxylan-derived pentasaccharide [136]. It is very likely that many more DAMPs released from the host CW generated during plant-pathogen interactions await identification.

The ability of different CW-derived oligomers to induce defense responses indicates that they share common signaling elements among them and also with microbial PAMPs [120]. CW-derived DAMPs, like PAMPs, are likely recognized at the cell surface by pattern recognition receptors (PRRs) complexes, comprising receptor-like kinases and receptor like proteins acting as receptors and co-receptors for their ligands [137]. OG perception appears to be mediated by wall-associated kinases (WAKs), receptor-like kinases with an ectodomain featuring epidermal growth factor-like repeats [138,139], as a domain swap approach revealed that the Arabidopsis WAK1 perceives OGs and activates downstream responses [140]. The perception system of other CW-derived elicitors is still largely unknown. Qualitative and quantitative differences in the responses triggered by different CW-derived DAMPs might reflect differences in their perception system, or in their homeostasis. For instance, OGs, xyloglucan oligosaccharides and cellobiose induce in Arabidopsis the phosphorylation of the MAPKs MPK3 and MPK6, which are likely responsible for the downstream expression of defense responses [132,134,141,142]. Arabidopsis transcriptome profiles are very similar after cellobiose or OG treatment [132,143]. However, in contrast to OGs [141,142], cellobiose and xyloglucan oligosaccharides do not stimulate ROS production or callose deposition [132,134]. Indeed, complex regulatory events must modulate CW-derived DAMP activity, to ensure activation of appropriate defense-related mechanisms without excessive fitness cost. Moreover, since CW remodeling also occurs during growth and developmental processes, it is likely that CW-derived fragments with DAMP activity are released to low levels also in the absence of pathogens. Therefore, regulation of their activity must be fine-tuned to avoid unnecessary activation of defense responses. In the case of OGs, at least two separate mechanisms may contribute to their in vivo activity, acting on the transduction events as well as on the levels of active elicitors present in the apoplast. WAK1 forms a complex with the cytoplasmic plasma membrane-localized kinase-associated protein phosphatase KAPP and the glycine-rich protein GRP-3, which both negatively regulate OG-triggered expression of defense genes and production of ROS, and also affect basal resistance against *B. cinerea* [144]. Oxidation of OGs, mediated by recently identified Arabidopsis proteins, named OG-OXIDASEs (OGOXs), abolishes their activity [145]. OGOXs belong to the family of the berberine bridge enzyme-like proteins, a subgroup of flavin adenine dinucleotide-linked oxidases that are structurally characterized by a typical fold observed initially for vanillyl-alcohol oxidase [146]. Paradoxically, plants overexpressing OGOX1 are more resistant to *B. cinerea*, possibly because oxidized OGs are less sensitive to degradation by fungal PGs or as a consequence of H_2_O_2_ released as a by-product of OGOX activity [145]. These results indicate that OGOXs might fine-tune resistance to pathogens through multiple mechanisms that involve dampening of elicitor activity, generation of ROS, and modification of substrates for microbial CWDEs. More recently, another Arabidopsis berberine bridge enzyme-like protein, CD OXIDASE (CELLOX), was shown to have oxidase activity on CDs with DP3-6 [133]. Oxidized CDs also lose their eliciting activity and are less easily assimilated by fungi, and, as in the case of OGOX1, plants overexpressing CELLOX display enhanced resistance to *B. cinerea* [133].

### 3.2. CWI Surveillance Mechanisms and Plant Innate Immunity

Most of our understanding of the plant CWI maintenance mechanisms derives from studies conducted on plants subjected to CW damage artificially induced by chemical or genetic means. For instance, defects in cellulose biosynthesis caused by the Arabidopsis *constitutive expression of VSP1* (*cev1*)mutation results in the constitutive production of JA and ethylene, and ectopic lignin deposition [147,148]. Similarly, Arabidopsis seedlings treated with the herbicide isoxaben, which inhibits CESA activity, show accumulation of JA, salicylic acid (SA), and ROS, ectopic lignin and callose deposition, and upregulation of defense genes [149]. These responses are largely suppressed by osmotica like sorbitol or mannitol [149], suggesting that CW deformation and membrane stretching caused by the inability of the cell to withstand elevated turgor pressures act as signals of altered CWI. The CW, plasma membrane, and cytoskeleton form a continuum through the plant cell surface [150], regulating plasma membrane nanodomains, which are important for the localization of transport and signaling proteins and for their interactions [151] and might be important for sensing and responding to an increased plasma membrane tension caused by changes in osmotic pressure. A multiplicity of membrane proteins, including stretch-activated calcium channels and membrane proteins connecting the cytoskeleton to the CW, might contribute to perception of turgor-dependent signals generated by altered CWI. One potential candidate is the Arabidopsis plasma membrane calcium channel MID1-COMPLEMENTING ACTIVITY1 (MCA1), which is implicated in mechanical and hypo-osmotic stress perception [152] and is required for responses to altered CWI [153]. Extensive degradation of the plant CW caused by microbial CWDEs during infection can be envisioned to cause a sudden expansion of the plasma membrane, generating the abovementioned turgor-dependent signals that trigger compensatory responses to reinforce the CW. Treatments of Arabidopsis seedlings with a cocktail of CWDEs induce isoxaben, callose, and lignin deposition and JA and SA accumulation, and these responses are suppressed by sorbitol cotreatment [153]. Since cellulose is the major load-bearing component of the CW, it is expected that cellulases are mostly responsible for these turgor-mediated responses. Xylanase alone does not induce SA and JA production in Arabidopsis seedlings, whereas cellulase induces only SA accumulation [153]. Notably, pectinases can induce both SA and JA in an osmosensitive manner, and a combination of both cellulase and pectinase causes accumulation of even greater levels of these hormones [153], supporting the hypothesis that pectin degradation facilitates cellulose accessibility to hydrolytic enzymes.

Plant responses to loss of CWI are also mediated by specific membrane receptors, mostly belonging to the *Catharanthus roseus* Receptor Like Kinase 1-Like (*Cr*RLK1L) family [154], named after the species in which its first member (*Cr*RLK1) was identified [155]. Members of the *Cr*RLK1L subfamily have two extracellular regions with similarity to the putative carbohydrate-binding malectin domain, a transmembrane domain, and an intracellular kinase domain [156]. One Arabidopsis member of this family, THESEUS1 (THE1), was identified in a screen for suppressors of the short hypocotyl and ectopic lignin phenotype of a mutant impaired in the cellulose synthase subunit CESA6 [157]. THE1 seems to act as a sensor of CWI, putatively binding CW polysaccharides with its malectin-like domains [157]. Notably, THE1 appears to perceive CW modifications mediated by necrotrophic fungi, and it positively controls resistance to *B. cinerea* [158]. Engelsdorf et al. [153] reported that THE1 acts in the same pathway of MCA1 to stimulate the CWI maintenance system, as JA, SA, and lignin levels in isoxaben-treated *the1-1 mca1* seedlings were similar to those measured in the single mutants. Interestingly, the gain of function *the1-4* mutant shows enhanced responses to isoxaben, that are partially reduced in *the1-4 mca1* and *the1-4 fei2* seedlings suggesting that MCA1 and FEI2 act downstream from THE1 [153]. Another Arabidopsis *Cr*RLK1L, FERONIA (FER), affects cell growth and plays important roles in plant physiology, as suggested by the pleiotropic phenotype of *fer* mutants [159]. The extracellular domain of FER binds pectin in vitro and contributes to maintenance of CWI during salt stress [160], suggesting that this protein acts as a sensor of pectin integrity, though this function in vivo still needs to be demonstrated. Moreover, it is not known whether FER is also able to bind to OGs and mediate responses to these elicitors.

It is increasingly evident that the plant CWI maintenance system interacts in a complex way with the innate immune system to fine-tune defense responses, and that positive and negative crosstalk exists between CWI and PTI. Arabidopsis elicitor peptides (AtPeps) are endogenous elicitors which are perceived by PEP-RECEPTOR1 and 2 (PEPR1 and PEPR2) and contribute to immunity against bacteria and fungi [161]. Notably, CW damage stimulates the production of AtPep1 and AtPep3, which in turn repress some responses induced by altered CWI [153]. This suggests that AtPep-mediated signaling, which positively regulates PTI, can suppress defense responses controlled by the CWI maintenance system [153]. On the other hand, defense responses controlled by the CWI maintenance system might compensate to some extent for the loss of PTI signaling elements, since loss-of-function mutants for PEPR1 and PEPR2, as well as the PRR co-receptor BRASSINOSTEROID INSENSITIVE 1-ASSOCIATED KINASE 1 (BAK1), show enhanced JA and SA accumulation in response to isoxaben [153]. Increasing evidence indicates that FER, beside functioning as a pectin integrity sensor, can positively and negatively regulate plant immune responses. FER acts as a scaffold for PRRs, positively modulating immunity [162], and can interact with several RAPID ALKALINIZATION FACTORs (RALFs) [162,163]. RALFs are a family of cysteine-rich peptides shown to regulate growth and responses to abiotic and biotic stress [164]. In particular, perception of the Arabidopsis RALF1 and RALF23 by FER inhibits PTI [162]. In addition, FER can negatively regulate JA-mediated defense responses [165].

The exact contribution of the CWI surveillance systems in plant–pathogen interactions is still not clear, though modifications of CW composition or structure obtained by mutations in genes involved in the biosynthesis or modification of specific CW components can provide some hints. An altered CW might affect the ability of some pathogens to degrade it during host colonization, and/or lead to the activation of host defense responses [8,166,167]. Several mutants, or transgenic plants with alterations in CW composition, display constitutive activation of defense responses and enhanced resistance to some pathogens [8,9,10,11]. For instance, Arabidopsis plants with mutations in genes encoding the CESA subunits CESA4/IRREGULAR XYLEM5 (IRX5) and CESA8/IRX1, involved in cellulose deposition in secondary CWs, show enhanced resistance to *Ralstonia solanacearum*, *Plectosphaerella cucumerina*, *B. cinerea,* and *Erysiphe cichoracearum*, whereas mutations in CESA3/ISOXABEN RESISTANT1 (IXR1)/ CEV1 and CESA6/IXR2/PROCUSTE 1 (PRC1), required for cellulose biosynthesis in primary CWs, affect only susceptibility to *B. cinerea* and *E. cichoracearum* [147,168,169]. In addition, *cev1* mutants constitutively express JA- and ethylene-dependent defense responses [147,168], whereas resistance to *P. cucumerina* or *R. solanacearum* in *irx1* and *irx5* mutants is independent of JA but requires abscisic acid (ABA) [169]. Arabidopsis and tobacco plants expressing an *A. niger* PG show a reduction of de-esterified HG content [170], accumulate high levels of ROS and POD activity and display a strong resistance to *B. cinerea* [171]. Notably, these plants do not show a constitutive expression of marker genes typically induced by SA, ethylene or JA [172]. These results suggest that different signaling pathways are involved in the resistant phenotype of CW-related mutants, depending on the altered CW component, the cell types that are most affected by the alteration and/or the inoculated pathogen. Compensatory responses activated by loss of CWI might modify other CW components not directly affected by the mutation, adding complexity to the interpretation of the obtained results. For instance, growth defects in the Arabidopsis *quasimodo2-1* (*qua2-1*) mutant, impaired in HG biosynthesis [173], are partially suppressed by loss of the POD AtPRX71, which negatively regulates cell expansion in response to CW damage [172]. Similarly, cell adhesion defects in *qua2-1* can be restored by mutations in the *ESMERALDA1* (*ESMD1*) gene, encoding a putative *O*-fucosyltransferase possibly involved in the glycosylation of membrane proteins, without affecting pectin composition [174]. It is likely that the identification of additional suppressors of different defects caused by alterations in specific CW components might provide insights into the molecular mechanisms modulating defense and growth responses triggered by CW degradation. Moreover, a detailed characterization of a large set of CW-related mutants will be instrumental to understand the role of specific CW components in plant–pathogen interactions, and to determine the impact of their degradation on the outcome of an infection. Recently, this approach was used to identify correlations between the amounts of specific carbohydrates in the CW of Arabidopsis and susceptibility to different pathogens [175]. Notably, the same paper provides evidence that pectin-enriched CW fractions isolated from Arabidopsis CW-related mutants can induce immune responses in other plants, suggesting that an increased production of CW-derived DAMPs might contribute to their resistant phenotype [175]. Intriguingly, an Arabidopsis mutant for the UDP-glucuronate 4-epimerases GAE1 and GAE6, defective in pectin biosynthesis, is more susceptible to *B. cinerea* infection, possibly because of an impaired production of pectin-derived DAMPs [176].

## 4. Conclusions

In the past four decades, we have accumulated an overwhelming amount of evidence indicating that a key feature of most plant–pathogen interactions is the deconstruction of the host CW, where a significant portion of the evolutionary arms race between plants and their pathogens takes place (Figure 1). However, we are still far from having a complete view of the exact contribution of the degradation of the different CW structural components affects specific interactions, how plant cells perceive changes in CWI and mount appropriate adaptive responses, and how these responses influence the outcome of an infection, both through the modification of the CW itself and through the modulation of defense-related signaling pathways. Several questions are still open, as highlighted in a recent review [10]; among these, the most relevant for the field of plant–pathogen interactions are probably: (1) How many CW-derived elicitors exist and what role do they play in immunity? (2) What is the relationship between CWI maintenance, development, and immunity? It is expected that the adoption of novel tools and techniques to probe and image in vivo specific CW epitopes, and to analyze CW composition on a microscopic scale [10] will reveal so far undetected details of the complex events occurring in the plant CW during pathogen penetration and invasion. Most of the available knowledge derives from studies on the model organism Arabidopsis, but CW structure varies among plant species, and very little is known about CW-derived DAMPs and mechanisms of CWI surveillance in plants belonging to different taxonomic groups, most notably *Poaceae*, which contain mostly hemicellulosic glucuronoarabinoxylans but have little pectin [11]. This knowledge will be instrumental for the development of innovative technologies for crop protection and for breeding of new, durable resistances with limited cost to productivity, and it will also contribute to obtaining plant varieties improved for biomass conversion, uncoupling the targeted modification of CW structural components from its effects on plant growth and fitness.

## Abbreviations

ABA	Abscisic acid
AtPep	Arabidopsis elicitor peptide
Ara	Arabinose
CAZymes	Carbohydrate-active enzymes
CrRLK1L	Catharanthus roseus Receptor Like Kinase 1-Like
CW	Cell wall
CWI	Cell wall integrity
CWDE	Cell wall-degrading enzyme
CBH	Cellobiohydrolase
CD	Cellodextrin
CELLOX	Cellodextrin oxidase
CESA	Cellulose synthase
DAMP	Damage-Associated Molecular Patterns
DP	Degree of polymerization
GalA	D-Galacturonic acid
Glc	D-Glucose
Xyl	D-Xylose
EG	Endoglucanase
Fuc	Fucose
GAE	Glucuronate 4-epimerase
HG	Homogalacturonan
HRGP	Hydroxyl-proline-rich glycoprotein
JA	Jasmonate
LRR	Leucine-rich repeat
Rha	L-Rhamnose
Man	Mannose
OG	Oligogalacturonide
OGOX	Oligogalacturonide oxidase
PAMP	Pathogen-Associated Molecular Pattern
PRR	Pattern recognition receptor
PTI	Pattern-Triggered Immunity
PL	Pectate lyase
PNL	Pectin lyase
PME	Pectin methylesterase
PMEI	Pectin methylesterase inhibitor
PEPR	PEP Receptor
POD	Peroxidase
PG	Polygalacturonase
PGIP	Polygalacturonase-inhibiting protein
RALF	RAPID ALKALINIZATION FACTOR
ROS	Reactive oxygen species
RGI	Rhamnogalacturonan I
RGII	Rhamnogalacturonan II
Rha	Rhamnose
SA	Salicylic acid
TAXI	*T. aestivum* xylanase inhibitor
TLXI	Thaumatin-like xylanase inhibitor
WAK	Wall-associated kinase
XIP	Xylanase-inhibiting protein
XGA	Xylogalacturonan
XGH	Xylogalacturonan hydrolase
XEGIP	Xyloglucan-specific endoglucanase inhibitor protein
